# Trends in Term Intrapartum Stillbirth in Norway

**DOI:** 10.1001/jamanetworkopen.2023.34830

**Published:** 2023-09-27

**Authors:** Gulim Murzakanova, Sari Räisänen, Anne Flem Jacobsen, Branka M. Yli, Tiril Tingleff, Katariina Laine

**Affiliations:** 1 Department of Obstetrics, Oslo University Hospital, Oslo, Norway; 2 Institute of Clinical Medicine, Faculty of Medicine, University of Oslo, Oslo, Norway; 3 Tampere University of Applied Sciences, Tampere, Finland; 4 Norwegian Research Centre for Women’s Health, Oslo University Hospital, Oslo, Norway

## Abstract

**Importance:**

Fetal death during labor at term is a complication that is rarely studied in high-income countries. There is a need for large population-based studies to examine the rate of term intrapartum stillbirth in high-income countries and the factors associated with its occurrence.

**Objective:**

To evaluate trends in term intrapartum stillbirth over time and to investigate the association between the trends and term intrapartum stillbirth risk factors from 1999 to 2018 in Norway.

**Design, Setting, and Participants:**

This cohort study used data from the Medical Birth Registry of Norway from 1999 to 2018 to examine rates of term intrapartum stillbirth and risk factors associated with this event. A population of 1 021 268 term singleton pregnancies without congenital anomalies or antepartum stillbirths was included in analyses, which were performed from September 2022 to February 2023.

**Exposure:**

The main exposure variable was time, which was divided into four 5-year periods: 1999 to 2003, 2004 to 2008, 2009 to 2013, and 2014 to 2018.

**Main Outcomes and Measures:**

The primary study outcome was term intrapartum stillbirth. Risk ratios were calculated, and multivariable logistic regression analyses were conducted to identify factors associated with secular trends of term intrapartum stillbirth.

**Results:**

The study population consisted of 1 021 268 term singleton births (maternal mean [SD] age, 29.72 [5.01] years; mean [SD] gestational age, 39.69 [1.27] weeks). During the study period, there were 95 term intrapartum stillbirths (0.09 per 1000 births). Maternal age, the proportion of individuals born in a country other than Norway, and the prevalence of gestational diabetes, labor induction, operative vaginal delivery, and previous cesarean delivery increased over the course of the study period. Conversely, the prevalence of infants large for gestational age, hypertensive disorder in pregnancy, and spontaneous vaginal delivery and the proportion of individuals who smoked decreased. The term intrapartum stillbirth rate decreased by 87% (95% CI, 68%-95%) from 0.15 per 1000 births in 1999 to 2008 to 0.02 per 1000 births in 2014 to 2018. Three in 4 term intrapartum stillbirths (70 of 95) occurred during intrapartum operative deliveries. The increased prevalence of older maternal age and obstetric risk factors were not associated with the variation in intrapartum stillbirth rates among the time periods. The prevalence of term intrapartum stillbirth was higher for individuals who gave birth in maternity units with fewer than 3000 annual births (adjusted odds ratio, 1.67; 95% CI, 1.07-2.61) than for those who gave birth in units with 3000 or more annual births.

**Conclusions and Relevance:**

Findings of this study suggest that, despite increases in maternal and obstetric risk factors, term intrapartum stillbirth rates substantially decreased during the study period. Reasons for this decrease may be due to improvements in intrapartum care.

## Key Points

**Question:**

How did the term intrapartum stillbirth rate change in Norway from 1999 to 2018?

**Findings:**

This cohort study of 1 021 268 births found an 87% reduction in term intrapartum stillbirth rates from 1999 to 2018. This decline was not associated with an increasing prevalence of at-risk pregnancies during the study period.

**Meaning:**

Findings of this study suggest that the incidence of term intrapartum stillbirth substantially decreased during the study period, possibly due to improvements in intrapartum care.

## Teaser

This cohort study examines term intrapartum stillbirth rates in Norway from 1999 to 2018.

## Introduction

Stillbirth is defined as a fetal death that occurs either during pregnancy at a gestational age (GA) of 22 weeks or later (antepartum stillbirth) or during labor (intrapartum stillbirth). Stillbirth can psychosocially affect both parents and health care workers and imposes an economic burden on society.^1,2,3^ Stillbirth rates in low- and middle-income countries are high.^4,5^ In high-income countries, a notable proportion of stillbirths (20%-30%) may occur due to substandard care, thus being preventable.^6,7^ The risk factors for intrapartum stillbirth may differ between high- and low-income countries.^8^ In high-income countries, constantly decreasing fertility rates and postponement of first birth have been observed, and individuals giving birth at a more advanced age has resulted in an increasing number of at-risk pregnancies.^9,10^ Increased rates of cesarean delivery have also been reported globally.^11^

Only a few studies have assessed time trends in intrapartum stillbirth rates in high-income countries, and their populations included preterm births, multifetal births, and infants with congenital malformations.^12,13^ Some studies combined intrapartum stillbirth with neonatal mortality as an outcome,^14^ while others compared intrapartum stillbirth rates between midwife- and obstetrician-led care.^15,16^ A 2011 systematic review^17^ included population-based studies from high-income countries that addressed stillbirth risk factors. However, the population of most of the studies included in that review consisted of preterm births, and stillbirths were not stratified into antepartum and intrapartum.

Few studies have assessed intrapartum stillbirth in term deliveries in high-income countries, probably due to its rarity. Many countries record data only as perinatal mortality or stillbirth, and such registries cannot identify intrapartum stillbirths. There is a need for large, population-based studies in high-income countries that investigate term intrapartum stillbirths. The aims of this study were to determine trends of term intrapartum stillbirth over time and to investigate the association between the trends and term intrapartum stillbirth risk factors from 1999 to 2018 in Norway.

## Methods

### Study Design and Participants

This population-based cohort study was conducted as a part of the PURPLE Study, which assesses pregnancy and birth outcomes in Norway.^18,19,20,21^ The present study was approved by the Regional Committee for Medical Research Ethics in South-East Norway and the Institutional Personal Data Officer at Oslo University Hospital. Patients were not involved in the planning, design, or conduct of this study. Data were collected in accordance with the Norwegian Health Research Act from the Medical Birth Registry of Norway (MBRN), which was established in 1967 and includes information on all births in Norway. The MBRN is a mandatory national health registry that does not require informed consent for the registration or use of data for research purposes. The study followed the Strengthening the Reporting of Observational Studies in Epidemiology (STROBE) reporting guideline.

The study population included births registered starting in 1999 because that was when the MBRN began defining GA based on second-trimester ultrasonography. Births with fetal congenital anomalies, preterm births (GA <37 weeks), multifetal births, antepartum stillbirths, home births, and transport births were excluded. Births with an estimated GA of 45 weeks or greater were also excluded on the assumption that these records were probably due to incorrect GA estimation. The study population consisted of 1 021 268 singleton births with GAs from 37 weeks to 44 weeks and 6 days. The study duration was divided into the following four 5-year periods to track possible changes in term intrapartum stillbirth rates: 1999 to 2003, 2004 to 2008, 2009 to 2013, and 2014 to 2018. Intrapartum stillbirth rates were calculated and presented per 1000 births.

### Outcome and Exposure Variables and Variable Definitions

The main outcome measure was term intrapartum stillbirth, which was defined as intrauterine fetal death during labor after confirmation of the presence of a fetal heartbeat at the onset of labor. The main exposure variable was time period.

Maternal age was categorized into 4 age groups: 24 years or younger, 25 to 29 years, 30 to 34 years, and 35 years or older. Maternal country of birth was categorized as Norway or other (all countries other than Norway). Maternal smoking status was categorized as no, sometimes or daily, and missing information. Hypertensive disorders in pregnancy included either gestational hypertension (blood pressure >140/90 mm Hg after 20 weeks’ GA), preeclampsia (blood pressure >140/90 mm Hg and proteinuria after 20 weeks’ GA), HELLP syndrome (hemolysis, elevated liver enzymes, low platelet count), or eclampsia (grand mal seizures during pregnancy, delivery, or within 7 days postpartum in individuals with gestational hypertension, preeclampsia, or HELLP syndrome). Maternal type 1, type 2, and gestational diabetes were categorized as no or yes. Placental abruption was defined as either partial or complete placental detachment prior to fetus delivery. Small for GA was defined as 5th percentile or less in birthweight and large for GA (LGA) as 90th percentile or greater according to Norwegian fetal growth statistics.^22^ Meconium-stained amniotic fluid was defined as foul-smelling or discolored amniotic fluid after membrane rupture. Prelabor rupture of membranes included all individuals with membrane rupture 12 hours or more before birth.

Maternity units were dichotomized by the number of annual births (<3000 or ≥3000) to determine whether maternity unit size was associated with term intrapartum stillbirth rates. An at-risk pregnancy was defined as individuals with at least 1 of the following conditions: maternal age 35 years or older, hypertensive disorder in pregnancy, diabetes (type 1 or 2 or gestational), previous cesarean delivery, or labor induction. Individuals without these conditions were defined as nonrisk pregnancies. Intrapartum operative delivery was defined as a delivery either by vacuum extraction, forceps, or emergency cesarean delivery during labor or delivery.

### Statistical Analysis

When there were fewer than 5 individuals in a category defined by descriptive statistics, the numbers were not reported to protect the rights of the involved individuals according to the General Data Protection Regulation of the European Union.^23^ Univariable and multivariable logistic regression analyses were performed to determine crude odds ratios (ORs) and adjusted ORs (AORs) with 95% CIs, and a backward stepwise approach was used to select variables associated with term intrapartum stillbirth. The multivariable logistic regression model included the following variables: time period, maternity unit size, maternal age, parity, labor induction, small for gestational age, LGA, meconium-stained amniotic fluid, previous cesarean delivery, placental abruption, and delivery type.

Risk ratios (RRs) with 95% CIs were calculated to assess for an association between term intrapartum stillbirth rates in at-risk pregnancies compared with nonrisk pregnancies, and to assess for an association between term intrapartum stillbirth rates in intrapartum operative deliveries compared with the reference group (spontaneous deliveries or planned cesarean delivery). Planned cesarean delivery was considered a low risk for term intrapartum stillbirth and was therefore merged with spontaneous delivery in the reference group.

To assess the association between maternal and obstetric risk factors and the changes in the term intrapartum stillbirth rates among time periods, we performed a logistic regression analysis with 3 different models. In model 1 the crude analysis was adjusted for maternal age, model 2 was adjusted for at-risk pregnancy, and model 3 was adjusted for intrapartum operative delivery. The following formula was used to calculate the percentage reduction in ORs between time periods: (OR Crude Model – Adjusted OR Model *x*)/(OR Crude Model – 1), where *x* was 2 or 3,^24^ if applicable. Values greater than 0 were considered valid contributions. Additionally, sensitivity analyses were performed to examine whether analyzing term intrapartum stillbirths together with neonatal mortality within 24 hours after delivery would alter the results of the main analyses.

Data were analyzed between September 2022 and February 2023 using SPSS Statistics, version 28 (IBM). A missing information category was created for the smoking variable, which was analyzed separately. The missing information rate was less than 1% for all other variables.

## Results

### Maternal Demographic Characteristics and Obstetric Outcomes

The study population consisted of 1 021 268 term singleton births (maternal mean [SD] age, 29.72 [5.01] years; mean [SD] gestational age, 39.69 [1.27] weeks). Population characteristics are shown in Table 1. During the study period, the proportion of individuals aged 35 years or older increased from 14.59% to 19.85%. The proportion of individuals born in a country other than Norway almost doubled (from 16.85% to 29.88%), and the prevalence of gestational diabetes increased 6-fold (from 0.74% to 4.56%). The incidence of labor induction doubled (from 10.60% to 21.54%), and the proportions of operative vaginal deliveries (from 8.09% to 10.55%) and previous cesarean delivery (from 7.94% to 9.72%) also increased. Conversely, there were decreases in the prevalence of LGA (from 13.24% to 9.89%), hypertensive disorder in pregnancy (from 4.78% to 3.61%), spontaneous vaginal delivery (from 79.86% to 75.00%), and the proportion of individuals who smoked (from 19.53% to 4.38%) (Table 1).

**Table 1.** Maternal Demographic Characteristics and Obstetric Outcomes Among 4 Time Periods and by Maternity Unit Size From 1999 to 2018 in Norway

	Time period, No. (%)				Annual No. of births in maternity unit, No. (%)	
	1999-2003 (n = 248 162)	2004-2008 (n = 251 408)	2009-2013 (n = 265 875)	2014-2018 (n = 255 823)	≥3000 (n = 422 605)	<3000 (n = 598 663)
**Demographic characteristics**						
Maternal age, y						
<20	6123 (2.47)	5499 (2.19)	5030 (1.89)	2737 (1.07)	5889 (1.39)	13 500 (2.26)
20-24	38 094 (15.35)	36 388 (14.47)	38 809 (14.60)	28 958 (11.32)	50 639 (11.98)	91 610 (15.30)
25-29	86 489 (34.85)	79 435 (31.60)	84 322 (31.72)	84 059 (32.86)	136 470 (32.29)	197 835 (33.05)
30-34	81 233 (32.73)	85 455 (33.99)	87 070 (32.75)	89 306 (34.91)	150 210 (35.54)	192 854 (32.21)
35-39	31 278 (12.60)	38 168 (15.18)	42 400 (15.95)	41 951 (16.40)	67 128 (15.88)	86 669 (14.48)
≥40	4945 (1.99)	6463 (2.57)	8244 (3.10)	8812 (3.45)	12 269 (2.90)	16 195 (2.71)
Maternal country of birth						
Norway	206 352 (83.15)	202 544 (80.56)	199 268 (74.95)	179 379 (70.12)	308 779 (73.07)	478 764 (79.97)
Other than Norway^a^	41 810 (16.85)	48 864 (19.44)	66 607 (25.05)	76 444 (29.88)	113 826 (26.93)	119 899 (20.03)
Smoking						
No	162 591 (65.52)	173 161 (68.88)	202 875 (76.30)	221 064 (86.41)	305 446 (72.28)	454 245 (75.88)
Sometimes or daily	48 459 (19.53)	31 778 (12.64)	23 585 (8.87)	11 223 (4.38)	36 162 (8.56)	78 883 (13.18)
Missing data	37 112 (14.96)	46 469 (18.48)	39 415 (14.82)	23 536 (9.20)	80 997 (19.17)	65 535 (10.95)
Nulliparity	98 998 (39.89)	104 023 (41.38)	111 816 (42.06)	107 499 (42.02)	185 158 (43.81)	237 178 (39.62)
**Outcomes**						
Hypertensive disorder in pregnancy^b^	11 874 (4.78)	11 892 (4.73)	10 819 (4.07)	9242 (3.61)	21 102 (4.99)	22 725 (3.80)
Placental abruption	567 (0.23)	470 (0.19)	429 (0.16)	403 (0.16)	765 (0.18)	1104 (0.18)
Diabetes						
Type 1	831 (0.33)	874 (0.35)	981 (0.37)	832 (0.33)	1561 (0.37)	1957 (0.33)
Type 2	452 (0.18)	793 (0.32)	858 (0.32)	822 (0.32)	1369 (0.32)	1556 (0.26)
Gestational	1827 (0.74)	2501 (0.99)	5429 (2.04)	11 662 (4.56)	9364 (2.22)	12 052 (2.01)
SGA	12 097 (4.87)	13 033 (5.18)	14 518 (5.46)	13 051 (5.10)	23 268 (5.51)	29 431 (4.92)
LGA	32 847 (13.24)	28 806 (11.46)	26 388 (9.92)	25 303 (9.89)	43 398 (10.27)	69 947 (11.68)
Meconium-stained amniotic fluid	44 075 (17.76)	42 918 (17.07)	42 031 (15.81)	45 187 (17.66)	74 746 (17.69)	99 465 (16.61)
PROM ≥12 h	25 022 (10.08)	32 591 (12.96)	40 491 (15.23)	44 808 (17.52)	64 741 (15.32)	78 171 (13.06)
Previous cesarean delivery	19 696 (7.94)	21 882 (8.70)	24 746 (9.31)	24 870 (9.72)	34 610 (8.19)	56 584 (9.45)
Induced labor	26 317 (10.60)	34 760 (13.83)	48 677 (18.31)	55 117 (21.54)	71 415 (16.90)	93 456 (15.61)
Delivery type						
Spontaneous vaginal delivery	198 186 (79.86)	193 583 (77.00)	200 104 (75.26)	191 871 (75.00)	321 496 (76.07)	462 248 (77.21)
Operative vaginal delivery	19 615 (8.09)	22 571 (9.18)	26 850 (10.13)	26 964 (10.55)	45 776 (10.83)	50 224 (8.40)
Intrapartum cesarean delivery	17 981 (7.13)	20 451 (8.07)	23 192 (8.72)	23 129 (9.03)	34 717 (8.16)	50 036 (8.32)
Planned cesarean delivery	12 380 (4.99)	14 803 (5.89)	15 729 (5.92)	13 853 (5.42)	20 616 (4.88)	36 155 (6.04)

Abbreviations: LGA, large for gestational age (>90th percentile); PROM, prelabor rupture of membranes; SGA, small for gestational age (<5th percentile).

^a^ All countries other than Norway.

^b^ Defined as gestational hypertension (blood pressure >140/90 mm Hg at >20 weeks’ gestational age), preeclampsia (blood pressure >140/90 mm Hg and proteinuria at >20 weeks’ gestational age), HELLP syndrome (hemolysis, elevated liver enzymes, low platelet count), or eclampsia (grand mal seizures during pregnancy, delivery, or within 7 days postpartum in individuals with gestational hypertension, preeclampsia, or HELLP syndrome).

### Trends in and Factors Associated With Term Intrapartum Stillbirth Rates

Term intrapartum stillbirth was observed in 0.09 per 1000 births (95 of 1 021 268 births) from 1999 to 2018. The rate decreased from 0.15 per 1000 births from 1999 to 2008 to 0.06 per 1000 births from 2009 to 2013 and to 0.02 per 1000 births from 2014 to 2018 (eTable 1 in Supplement 1; Figure). This represents an 87% (95% CI, 68%-95%) decrease in the term intrapartum stillbirth rate from 1999 to 2018.

**Figure.**
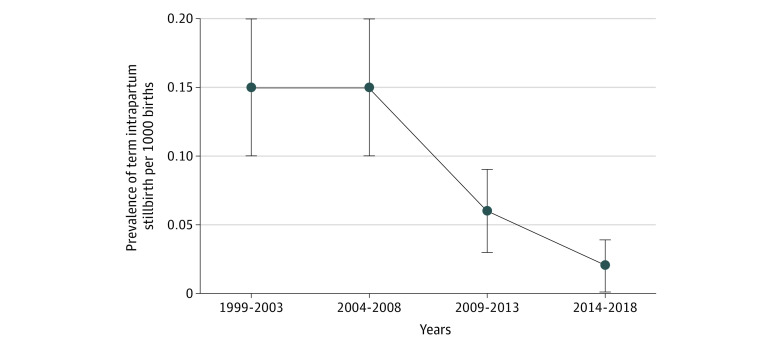
Term Intrapartum Stillbirth Rates in Norway From 1999 to 2018 The error bars represent 95% CIs.

The prevalence of term intrapartum stillbirth decreased by 87% (AOR, 0.13; 95% CI, 0.05-0.33) from 2014 to 2018 and by 62% (AOR, 0.38; 95% CI, 0.21-0.69) from 2009 to 2013 compared with the first time period (1999-2003) (Table 2). Table 3 shows that the risk of term intrapartum stillbirth was significantly higher in at-risk pregnancies compared with nonrisk pregnancies in the first 2 time periods: 0.27 vs 0.10 per 1000 births from 1999 to 2003 (RR, 2.78 [95% CI, 1.44-5.17]) and 0.25 vs 0.09 per 1000 births from 2004 to 2008 (RR, 2.89 [95% CI, 1.48-5.61]). The third period (2009 to 2013) and last period (2014 to 2018) showed no association between term intrapartum stillbirth rates and at-risk pregnancy status.

**Table 2.** Crude and Adjusted Odds Ratios for Term Intrapartum Stillbirth

Variable	Term intrapartum stillbirth	
	Crude analyses, OR (95% CI)	Multivariable logistic regression analyses, AOR (95% CI)
Time periods		
1999-2003	1 [Reference]	1 [Reference]
2004-2008	0.96 (0.61-1.51)	0.99 (0.63-1.56)
2009-2013	0.37 (0.20-0.67)	0.38 (0.21-0.69)
2014-2018	0.13 (0.05-0.32)	0.13 (0.05-0.33)
Maternity unit annual births		
≥3000	1 [Reference]	1 [Reference]
<3000	1.69 (1.09-2.63)	1.67 (1.07-2.61)
Maternal age, y		
≤24	0.69 (0.32-1.47)	NS
25-29	1 [Reference]	1 [Reference]
30-34	1.34 (0.81-2.19)	NS
≥35	1.50 (0.85-2.63)	NS
Parity		
Nulliparous	1 [Reference]	1 [Reference]
Parous	0.75 (0.50-1.12)	NS
Induced labor	1.76 (1.11-2.79)	NS
SGA	3.45 (1.99-5.98)	2.58 (1.46-4.56)
LGA	1.87 (1.12-3.13)	1.83 (1.08-3.11)
Meconium-stained amniotic fluid	4.57 (3.05-6.83)	3.39 (2.25-5.13)
Previous cesarean delivery	2.55 (1.54-4.22)	1.72 (1.02-2.90)
Placental abruption	87.07 (48.42-156.58)	23.44 (12.37-44.41)
Delivery type		
Spontaneous vaginal delivery	1 [Reference]	1 [Reference]
Operative vaginal delivery	8.52 (4.81-15.10)	7.94 (4.46-14.14)
Planned cesarean delivery	1.20 (0.28-5.09)	1.14 (0.26-4.98)
Intrapartum cesarean delivery	18.50 (11.22-30.53)	10.32 (6.02-17.70)

Abbreviations: AOR, odds ratio adjusted for all the variables in the table; LGA, large for gestational age; NS, nonsignificant; OR, odds ratio; SGA, small for gestational age.

**Table 3.** Risk Ratios (RRs) for Term Intrapartum Stillbirths in At-Risk Pregnancies Compared With Nonrisk Pregnancies

Time period	No. of cases/total No. (per 1000 births)		RR of term intrapartum stillbirth (95% CI)
	At-risk pregnancies^a^	Nonrisk pregnancies	
1999-2003	21/77 351 (0.27)	17/170 811 (0.10)	2.78 (1.44-5.17)
2004-2008	23/91 179 (0.25)	14/160 229 (0.09)	2.89 (1.48-5.61)
2009-2013	8/107 756 (0.07)	7/158 119 (0.04)	1.66 (0.61-4.62)
2014-2018	<5/112 395 (0.03)^b^	<5/143 428 (<0.01)^b^	1.94 (0.32-11.46)
Total	55/388 681 (0.14)	40/632 587 (0.06)	2.24 (1.49-3.36)

^a^ Individuals with at least 1 of the following: maternal age 35 years or older, hypertensive disorder in pregnancy, any diabetes, labor induction, or previous cesarean delivery. Nonrisk pregnancy was the reference category.

^b^ In groups with fewer than 5 cases the exact number is not shown to protect involved individuals’ privacy rights according to the European Union’s General Data Protection Regulation.

Most term intrapartum stillbirths (74% [70 of 95]) occurred during intrapartum operative deliveries (eTable 1 in Supplement 1). Table 4 shows that the risk of term intrapartum stillbirth decreased over time for both intrapartum operative deliveries and the reference group (spontaneous deliveries or planned cesarean delivery). The risk of term intrapartum stillbirth for intrapartum operative deliveries compared with the reference group was 10- to 28-fold higher; for example, in 2009 to 2013, there were fewer than 5 term intrapartum stillbirths in the reference group of 215 826 deliveries and 13 term intrapartum stillbirths among 50 049 intrapartum operative deliveries, for an RR of 28.03 (95% CI, 6.33-124.21). During the last time period (2014-2018), all 5 term intrapartum stillbirths occurred in intrapartum operative deliveries. In addition, individuals who gave birth in maternity units with fewer than 3000 annual deliveries had a 67% increased prevalence (AOR, 1.67; 95% CI, 1.07-2.61) of term intrapartum stillbirth compared with those who gave birth in units with 3000 or more annual deliveries (Table 2).

**Table 4.** Risk Ratios (RRs) for Term Intrapartum Stillbirth in Intrapartum Operative Deliveries Compared With Spontaneous Deliveries or Planned Cesarean Delivery

Time period	No. of cases/total No. (per 1000 births)		RR of term intrapartum stillbirth (95% CI)
	Intrapartum operative deliveries^a^	Spontaneous deliveries or planned cesarean delivery	
1999-2003	25/37 643 (0.66)	13/210 519 (0.06)	10.76 (5.50-21.02)
2004-2008	27/43 134 (0.62)	10/208 274 (0.05)	13.04 (6.31-26.93)
2009-2013	13/50 049 (0.26)	<5/215 826 (<0.01)^b^	28.03 (6.33-124.21)
2014-2018	5/50 095 (0.01)	0/0	NA
Total	70/180 921 (0.04)	25/840 347 (0.03)	13.01 (8.24-20.53)

Abbreviation: NA, not applicable.

^a^ Definition of intrapartum operative delivery: forceps, vacuum extraction, or intrapartum cesarean delivery. Spontaneous deliveries or planned cesarean delivery was the reference category.

^b^ In groups with fewer than 5 cases the exact number is not shown to protect involved individuals’ privacy rights according to the European Union’s General Data Protection Regulation.

The eTable 2 in Supplement 1 shows that, in all 3 logistic regression models, the AORs for term intrapartum stillbirth increased (compared with the crude analyses), suggesting that the variables in the models were not associated with the differences in term intrapartum stillbirth prevalence among the time periods. In sensitivity analyses, the same downward trends were observed when term intrapartum stillbirth was analyzed with neonatal mortality within 24 hours after delivery (eFigure and eTable 3 in Supplement 1).

## Discussion

This study used population-based registry data and found that the prevalence of term intrapartum stillbirth in Norway decreased from 0.15 to 0.02 per 1000 births (87%) between 1999 and 2018. The decline was greatest from 2009 to 2013 and continued to decrease throughout the last period (2014-2018). A significant decline in term intrapartum stillbirths was observed both in at-risk pregnancies (maternal age ≥35 years, hypertensive disorder in pregnancy, any diabetes, previous cesarean delivery, or labor induction) and in intrapartum operative deliveries. This reduction was observed in a period during which the proportion of individuals with at-risk pregnancies and intrapartum operative deliveries increased. Also, the incidence of labor induction, suggesting an at-risk pregnancy, increased significantly.

These reductions in term intrapartum stillbirth rates may be associated with improvements in intrapartum care. In this study, 3 of 4 term intrapartum stillbirths occurred in operative deliveries. We assume that many of these fetuses were delivered due to signs of hypoxia or asphyxia since threatening fetal asphyxia is 1 of the most common indications for operative delivery.^25,26^ While we did not obtain information about the direct causes of term intrapartum stillbirths, we believe that the 87% (95% CI, 68%-95%) rate reduction during the study period can be explained by improved intrapartum care and a decrease in hypoxia- or asphyxia-related fetal deaths. These results were consistent with those of an Irish study^13^ that found a decrease in hypoxia-related stillbirths in term singleton births from 1979 to 2003. Intrapartum fetal hypoxia or asphyxia is often caused by or coexists with term intrapartum stillbirth risk factors and can be detected using fetal heart rate (FHR) monitoring. Several improvements in intrapartum FHR monitoring were implemented in Norway during the study period.

A new computer-based intrapartum FHR monitoring method, ST waveform analyses of fetal electrocardiography (STAN), was first applied in Norway in 2002 following a randomized trial^27^ and a European observational study.^28^ Initially introduced to a few maternity units, the method was gradually adopted by several units nationwide. Before implementation of STAN, physicians and midwives were required to be educated and certified on the standardized interpretation of modified classification for intrapartum cardiotocography (CTG) from the International Federation of Gynecology and Obstetrics. This education was also provided in the remaining maternity units that have not adopted the STAN method. As a result, obstetricians and midwives throughout Norway used the same CTG classification, which likely was associated with an improved understanding and interpretation of CTG changes as well as better communication among health care professionals.

The Norwegian national guidelines for FHR monitoring during labor from 1998 contained inadequate guidance for CTG interpretation and adjunctive technology, such as fetal blood sampling (FBS). This national guideline, which is trusted and implemented among Norwegian professionals, was updated in 2008^29^ with a thorough explanation of the STAN methods and FBS as well as the physiological mechanisms underlying CTG patterns during labor. From 2009 to 2018, FBS was changed from pH to lactate analyses after a randomized clinical trial found that these methods were comparable.^30^ The blood sample required is larger in FBS for pH analysis (35-50 μL) than for lactate analysis (5 μL), and the latter is therefore more likely to be obtained.^31^

Continuous education on emergency obstetrics, fetal monitoring, and neonatal resuscitation was implemented in Norwegian maternity units due to requirements from the health authorities. Maternity units in Norway have established local perinatal audit groups that analyze all perinatal deaths regularly to identify potentially preventable factors, with the focus to learn from the events and improve care. Severe adverse events are reported to the Norwegian Board of Health Supervision, and maternity units involved are required to present an improvement plan to avoid such adverse events in the future.

The decline in term intrapartum stillbirth rates after 2008 may have been associated with all of the previously mentioned changes that were gradually implemented during the study period, contributing to continuous improvements in intrapartum care in Norway. Another relevant issue is that the largest decline in term intrapartum stillbirth rates found in the present study coincided with decreased overall cesarean delivery rates after 2008, as found in a recent Norwegian study^10^ that concluded that low cesarean delivery rates did not impair perinatal health. The findings of our study support this conclusion.

We found that the prevalence of term intrapartum stillbirth was higher for fetuses born in smaller maternity units than for those born in larger maternity units. However, we could not classify hospitals into more than 2 categories due to the low number of term intrapartum stillbirths (n = 95). The low number of cases in subgroups would have caused challenges related to personal data protection and statistical methods. This finding was consistent with those of previous Norwegian studies^32,33,34^ that assessed outcomes with a higher prevalence, namely perinatal and neonatal mortality, and found an increased prevalence of mortalities in smaller maternity units. The increased prevalence of term intrapartum stillbirth in the units with fewer than 3000 annual births could be due to geographic challenges in Norway; many individuals who live in rural areas travel a longer distance to a maternity unit. Experienced obstetricians are on call at larger maternity units, whereas they may be only on call at home in smaller units, which may be a risk factor according to a Finnish study.^35^ This finding warrants further investigation to better understand the factors contributing to differences in rates between unit sizes. We believe our findings are generalizable to high-income countries with universal access to maternity care services.

### Limitations

This study has some limitations. There may have been recording errors and missing data due to the nature of the registry data. However, the outcome measure, term intrapartum stillbirth, is information that is quality controlled and double-checked by communication between the MBRN and the maternity units. This information is also carefully entered in several registries. Liveborn infants are registered as new residents of Norway, and stillborn infants are registered in Norway Vital Statistics.^36^ Additionally, information on FHR monitoring methods during delivery was not recorded in the MBRN.

The term intrapartum stillbirth rates in our study could have been influenced by early neonatal mortality, which can also be associated with intrapartum complications.^37^ However, results of our sensitivity analyses that combined term intrapartum stillbirths with neonatal mortality within 24 hours after delivery were not significantly different from those of the main analyses.

## Conclusions

This cohort study found that despite some unfavorable changes in maternal health over time, the term intrapartum stillbirth rate decreased by 87% between 1999 and 2018, with a marked decline after 2008. This reduction may be due to improvements in intrapartum care marked by a focus on continuous education for intrapartum fetal surveillance and obstetric emergencies.

## Notes

Supplement 1. **eTable 1.** Term Intrapartum Stillbirth Rates by Time Periods, Maternity Unit Size, Demographics, and Maternal and Obstetric Outcomes (N=1,021,268) During 1999 to 2018 in Norway **eTable 2.** Crude and Adjusted Odds Ratios for Term Intrapartum Stillbirth in Time Periods Adjusted for 3 Different Models: Maternal Age (1), At-Risk Pregnancy (2), and Intrapartum Operative Delivery (3) **eFigure.** Rates of Term Intrapartum Stillbirth Combined With Neonatal Mortality Within 24 Hours After Delivery in Norway, 1999 to 2018 **eTable 3.** Crude and Adjusted Odds Ratios for Term Intrapartum Stillbirth Combined With Neonatal Mortality Within 24 Hours After Delivery in Norway, 1999 to 2018 (N=1,021,268)

Supplement 2.
Data Sharing Statement
