# Errors in Table 4

**DOI:** 10.1001/jamanetworkopen.2024.0314

**Published:** 2024-01-31

In the Original Investigation titled “Trends in Term Intrapartum Stillbirth in Norway”^1^ published on September 27, 2023, there were errors in Table 4. The number of intrapartum deliveries per 1000 births from 2014 to 2018 should be 0.10. The total number of intrapartum deliveries per 1000 births should be 0.39. The article has been corrected.
