# Supplementary material for: Trends in Term Intrapartum Stillbirth in Norway
Source: JAMA Netw Open. 2023 Sep 27;6(9):e2334830. doi: 10.1001/jamanetworkopen.2023.34830 (PMC10534268; doi:10.1001/jamanetworkopen.2023.34830)
Supplement: Supplement 1. — eTable 1. Term Intrapartum Stillbirth Rates by Time Periods, Maternity Unit Size, Demographics, and Maternal and Obstetric Outcomes (N=1,021,268) During 1999 to 2018 in Norway eTable 2. Crude and Adjusted Odds Ratios for Term Intrapartum Stillbirth in Time Periods Adjusted for 3 Different Models: Maternal Age (1), At-Risk Pregnancy (2), and Intrapartum Operative Delivery (3) eFigure. Rates of Term Intrapartum Stillbirth Combined With Neonatal Mortality Within 24 Hours After Delivery in Norway, 1999 to 2018 eTable 3. Crude and Adjusted Odds Ratios for Term Intrapartum Stillbirth Combined With Neonatal Mortality Within 24 Hours After Delivery in Norway, 1999 to 2018 (N=1,021,268) [file jamanetwopen-e2334830-s001.pdf]

Supplemental Online Content

Murzakanova G, Räisänen S, Jacobsen AF, Yli BM, Tingleff T, Laine K. Trends in term intrapartum stillbirth in Norway. *JAMA Netw Open*. 2023;6(9):2334830. doi:10.1001/jamanetworkopen.2023.34830

eTable 1. Term Intrapartum Stillbirth Rates by Time Periods, Maternity Unit Size, Demographics, and Maternal and Obstetric Outcomes (N=1,021,268) During 1999 to 2018 in Norway

eTable 2. Crude and Adjusted Odds Ratios for Term Intrapartum Stillbirth in Time Periods Adjusted for 3 Different Models: Maternal Age (1), At-Risk Pregnancy (2), and Intrapartum Operative Delivery (3)

eFigure. Rates of Term Intrapartum Stillbirth Combined With Neonatal Mortality Within 24 Hours After Delivery in Norway, 1999 to 2018

eTable 3. Crude and Adjusted Odds Ratios for Term Intrapartum Stillbirth Combined With Neonatal Mortality Within 24 Hours After Delivery in Norway, 1999 to 2018 (N=1,021,268)

This supplemental material has been provided by the authors to give readers additional information about their work.

eTable 1. Term Intrapartum Stillbirth Rates by Time Periods, Maternity Unit Size, Demographics, and Maternal and Obstetric Outcomes (N=1,021,268) During 1999 to 2018 in Norway

	**Term intrapartum stillbirth**	**Live birth**
	No. (per 1000 births)	No.
Entire study population	95 (0.09)	1,021,173
**Time period**		
Time period 1 (1999–2003)	38 (0.15)	248,124
Time period 2 (2004–2008)	37 (0.15)	251,371
Time period 3 (2009–2013)	15 (0.06)	265,860
Time period 4 (2014–2018)	6 (0.02)	255,818
**Maternity unit size**		
≥3000 annual births	28 (0.07)	422,577
<3000 annual births	67 (0.11)	598,596
**Maternal age, years**		
≤24	9 (0.06)	161,629
25–29	27 (0.08)	334,278
30–34	37 (0.11)	343,027
≥35	22 (0.12)	182,239
**Maternal country of birth**		
Norway	79 (0.10)	787,464
Other than Norway[a]	16 (0.07)	233,709
**Smoking**		
No	67 (0.09)	759,624
Sometimes or daily	15 (0.13)	115,030
Missing information	13 (0.09)	146,519
**Parity**		
Nulliparous	46 (0.11)	422,290
Parous	49 (0.08)	598,883
**Hypertensive disorder in pregnancy**	8 (0.18)	43,819
**Placental abruption**	13 (7.00)	1,856
**Diabetes all types**	<5[b]	27,858
**SGA <5th percentile**	15 (0.29)	52,684
**LGA >90th percentile**	18 (0.16)	113,326
**Meconium–stained amniotic fluid**	46 (0.26)	174,165
**PROM≥12 hours**	20 (0.14)	142,892
**Previous CS**	19 (0.21)	84,152
**Induced labor**	24 (0.15)	164,847
**Delivery type**		
Spontaneous vaginal delivery	23 (0.03)	783,721
Operative vaginal delivery	24 (0.25)	95,976
Intrapartum CS	46 (0.54)	84,707
Planned CS	<5[b]	56,769

Abbreviations: SGA, small for gestational age; LGA, large for gestational age; PROM, prelabor rupture of membranes; CS, cesarean section.
[a] All countries other than Norway
[b] Cases less than 5 are not shown to protect involved individuals' right according to European Union's General Data Protection Regulation.

eTable 2. Crude and Adjusted Odds Ratios for Term Intrapartum Stillbirth in Time Periods Adjusted for 3 Different Models: Maternal Age (1), At-Risk Pregnancy (2), and Intrapartum Operative Delivery (3)

	Crude OR (95% CI)	Model 1[a] aOR (95% CI)	Model 2[b] aOR (95% CI)	Diff. from Crude[c] (%)	Model 3[d] aOR (95% CI)	Diff. from Crude[c] (%)
1999–2003	7.84 (3.08–19.91)	8.24 (3.24–20.94)	8.88 (3.49–22.58)	<0	9.35 (3.68–23.77)	<0
2004–2008	7.53 (2.96–19.16)	7.73 (3.04–19.67)	8.10 (3.18–20.62)	<0	8.27 (3.25–21.04)	<0
2009–2013	2.89 (1.05–7.94)	2.95 (1.07–8.13)	2.98 (1.08–8.20)	<0	2.97 (1.08–8.17)	<0
2014–2018	1 [Reference]	1 [Reference]	1 [Reference]		1 [Reference]	

Abbreviations: OR, odds ratio; CI, confidence interval; aOR, adjusted odds ratio.

[a] Model 1 was adjusted for maternal age.

[b] Model 2 was adjusted for risk pregnancy (at least one of the following conditions: maternal age ≥35, hypertensive disorder in pregnancy, any diabetes, labor induction or previous cesarean section).

[c] The percentage differences between Crude and Model 2; Crude and Model 3 were calculated by the formula (OR Crude Model– aOR Model x)/(OR Crude Model –1). x=aOR in Model 2 or Model 3.

[d] Model 3 was adjusted for intrapartum operative delivery (forceps, vacuum extraction, or intrapartum cesarean section).

eFigure. Rates of Term Intrapartum Stillbirth Combined With Neonatal Mortality Within 24 Hours After Delivery in Norway, 1999 to 2018

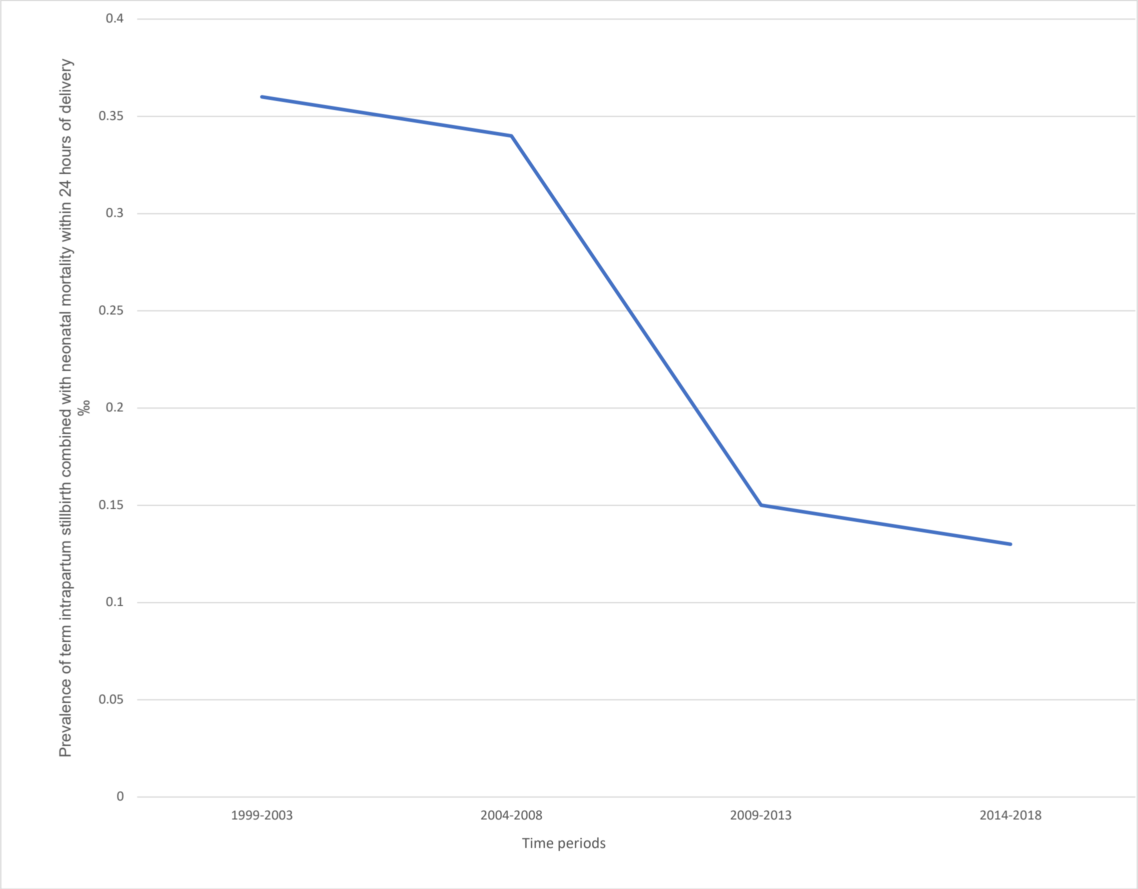

eTable 3. Crude and Adjusted Odds Ratios for Term Intrapartum Stillbirth Combined With Neonatal Mortality Within 24 Hours After Delivery in Norway, 1999 to 2018 (N=1,021,268)

**Variables**	**Term intrapartum stillbirth combined with neonatal mortality within 24 hours of delivery**	
	**Crude analyses**	**Multivariable logistic regression analyses**
	**OR (95% CI)**	**aOR (95% CI)**
**Time periods**		
Time period 1 (1999–2003)	1 [Reference]	1 [Reference]
Time period 2 (2004–2008)	0.95 (0.71–1.29)	0.93 (0.69–1.26)
Time period 3 (2009–2013)	0.44 (0.30–0.63)	0.42 (0.29–0.62)
Time period 4 (2014–2018)	0.38 (0.25–0.56)	0.35 (0.24–0.52)
**Maternity unit size**		
≥3000 annual births	1 [Reference]	1 [Reference]
<3000 annual births	1.35 (1.04–1.76)	1.35 (1.04–1.76)
**Maternal age, years**		
≤24	1.15 (0.76–1.75)	1.18 (0.77–1.80)
25–29	1 [Reference]	1 [Reference]
30–34	1.39 (1.00–1.93)	1.37 (0.98–1.91)
≥35	1.99 (1.40–2.81)	1.81 (1.26–2.58)
**Parity**		
Nulliparous	1 [Reference]	1 [Reference]
Parous	0.83 (0.65–1.06)	NS
**Induced labor**	1.59 (1.18–2.13)	NS
**SGA <5th percentile**	2.92 (2.03–4.20)	2.29 (1.58–3.33)
**LGA >90th percentile**	1.72 (1.25–2.39)	1.59 (1.13–2.22)
**Meconium–stained amniotic fluid**	3.40 (2.64–4.38)	2.49 (1.92–3.23)
**Previous CS**	2.21 (1.58–3.08)	
**Placental abruption**	45.71 (28.56–73.14)	11.38 (6.91–18.75)
**Delivery type**		
Spontaneous vaginal delivery	1 [Reference]	1 [Reference]
Operative vaginal delivery	5.70 (4.01–8.09)	6.19 (4.28–8.95)
Planned CS	1.27 (0.59–2.76)	1.32 (0.60–2.87)
Intrapartum CS	13.64 (10.20–18.26)	10.43 (7.61–14.29)

Abbreviations: OR, odds ratio; aOR, odds ratio adjusted for all the variables in the table; CI, confidence interval; NS, non-significant; SGA, small for gestational age; LGA, large for gestational age; CS, cesarean section.
