# Supplementary material for: Trends in Term Intrapartum Stillbirth in Norway
Source: JAMA Netw Open. 2023 Sep 27;6(9):e2334830. doi: 10.1001/jamanetworkopen.2023.34830 (PMC10534268; doi:10.1001/jamanetworkopen.2023.34830)
Supplement: Supplement 2. — Data Sharing Statement [file jamanetwopen-e2334830-s002.pdf]

Data Sharing Statement

Murzakanova. Trends in Term Intrapartum Stillbirth in Norway. *JAMA Netw Open*. Published September 20, 2023. doi:10.1001/jamanetworkopen.2023.34830

Data
Data available: No

Additional Information
Explanation for why data not available: Due to the sensitive nature of the data and according to Norwegian research legislation and regulations governing the Medical Birth Registry of Norway, data availability is restricted to licensed research groups.
